# Severe lower gastrointestinal bleeding caused by rectal Dieulafoy’s lesion: Case reports and literature review

**DOI:** 10.1097/MD.0000000000032031

**Published:** 2022-12-02

**Authors:** Ping Han, Yu Lei, Wei Hou, Nianjun Chen, Jingmei Liu, Dean Tian, Qiaozhen Guo, Wei Yan

**Affiliations:** a Department of Gastroenterology, Tongji Hospital, Tongji Medical College, Huazhong University of Science and Technology, Wuhan 430030, China.

**Keywords:** Case report, Dieulafoy’s lesion, endoscopic hemostasis, gastrointestinal hemorrhage, rectum

## Abstract

Dieulafoy’s lesion are relatively rare and can cause severe gastrointestinal bleeding. A Dieulafoy’s lesion is defined as an artery that erodes the overlying epithelium without the presence of an ulcer. Bleeding in Dieulafoy’s lesion predominantly involves the proximal stomach and upper gastrointestinal tract and is rarely observed in the lower gastrointestinal tract.

**Patient concerns:**

Case 1 was a 58-year-old woman complaining of sudden headache and vomiting who was diagnosed with subarachnoid hemorrhage. She underwent transcatheter embolization for intracranial aneurysm treatment but had an acute profuse hematochezia on the 11th day of admission. Case 2 was a 63-year-old man admitted to the respiratory intensive care unit because of fever with altered consciousness level for a week. He was diagnosed with advanced lung cancer that had metastasized to multiple organs one month prior. On the third day of admission, he had an attack of profuse hematochezia, and quickly developed shock and apathy.

**Diagnosis:**

Both patients were diagnosed with actively bleeding rectal Dieulafoy’s lesion by bedside emergency colonoscopy.

**Interventions:**

Endoscopic hemostatic clipping was performed in 2 patients.

**Outcomes:**

Hemostasis was successfully achieved in these 2 patients, and there was no recurrence of symptoms during follow-up.

**Conclusions:**

We propose that hemostatic clipping is one of the options in the treatment of rectal Dieulafoy’s lesions.

## 1. Introduction

Dieulafoy’s lesion is an uncommon but well-recognized cause of significant gastrointestinal bleeding. This lesion was initially described as a gastric aneurysm by Gallard in 1884^[1]^ but was later named after a French surgeon, Georges Dieulafoy, who characterized it as “Exulceratio simplex” in 3 patients as the cause of gastrointestinal bleeding.^[2]^ In the gastrointestinal tract, Dieulafoy’s lesion refers to the persistence of an abnormally large and tortuous primary arterial branch that extends in the submucosa without caliber loss, protruding through a small mucosal defect.^[3]^ Dieulafoy’s lesion accounts for 5% of all causes of acute gastrointestinal bleeding, and over 70% of the lesions occur in the proximal stomach, especially in the lesser curvature of the gastroesophageal junction.^[4]^ However, these lesions have also been reported in other parts of the gastrointestinal tract, such as the esophagus, duodenum, jejunum, and colon.^[5]^

Rectal Dieulafoy’s lesion was first reported by Franko et al.^[6]^ Currently, limited studies report that rectal lesions account for < 2% of all Dieulafoy’s lesions, making it an extremely rare cause of gastrointestinal bleeding.^[7]^The etiology of rectal Dieulafoy’s lesion is unknown, and major underlying disorders include hypertension, diabetes mellitus, and chronic kidney disease.^[8]^ Surgery or selective arterial embolization have been the main treatment methods for rectal Dieulafoy’s lesion in the past. However, these patients frequently experience serious complications and are at high risk for surgery or respiratory or circulatory failure, and cannot be transferred to the intervention room.^[9]^ Bedside colonoscopy is currently considered the first option for the management of gastrointestinal Dieulafoy’s lesions.^[9]^ Although the effectiveness of different emergent endoscopic treatments, including injection, coagulation, clipping, and band ligation, in the management of rectal Dieulafoy’s lesions has been demonstrated,^[8]^ there is no consensus on the optimal endoscopic hemostasis technique for rectal Dieulafoy’s lesions owing to the rarity of the disease. We report 2 cases of acute hemorrhage due to rectal Dieulafoy’s lesion that were both successfully treated by endoscopic hemostatic clipping. We also present a review of literature on rectal Dieulafoy’s lesion and the recent trends in its clinical features, pathogenesis, and endoscopic therapies.

## 2. Consent

This study was approved by the Ethics Committee of Tongji Hospital, Tongji Medical College, Huazhong University of Science and Technology. Informed written consent was obtained from the patient for publication of this case report and accompanying images.

## 3. Case presentation

### 3.1. Case 1

**Figure 1.**
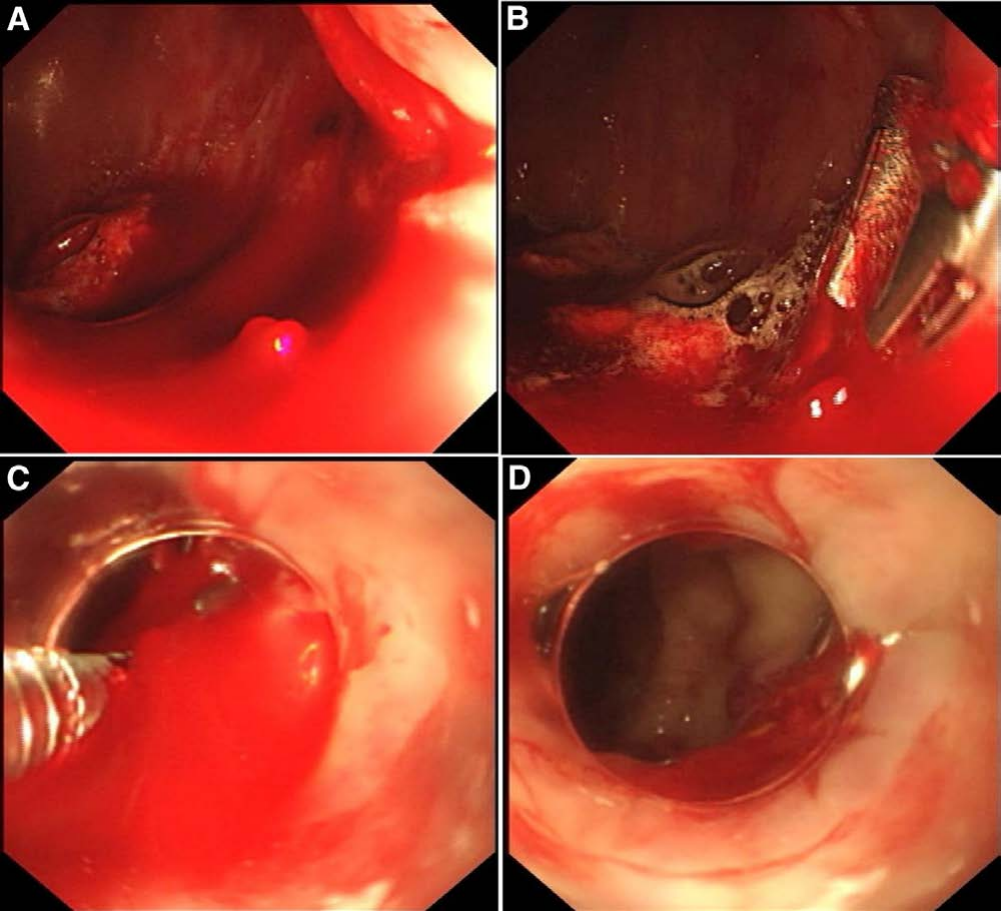
Endoscopy of patient 1. (A) Endoscopic view of a Dieulafoy’s lesion with pulsatile fresh bleeding in the rectum proximal to anal verge. (B) There was still active bleeding after 2 hemostatic clips placed in the lesion. (C) The third hemostatic clip was deployed under a transparent cap-assisted method. (D) Successful hemostasis was achieved with 3 hemostatic clips.

A 58-year-old woman complaining of sudden headache and vomiting was transferred to the neurosurgery intensive care unit of our hospital. She was diagnosed with subarachnoid hemorrhage by computed tomography. She had a history of hypertension and belonged to the Rh-negative blood group. Transcatheter embolization of intracranial aneurysms was performed on the day of admission. She received oral aspirin and clopidogrel bisulfate after the operation. The patient was repeatedly administered diclofenac sodium suppositories and intravenous injection of dexamethasone due to postoperative persistent fever. On the 11th day of admission, the patient had an acute profuse hematochezia and a decreased blood pressure of 74/54 mm Hg. Her hemoglobin level decreased from 11 g/dL at admission to 9 g/dL (normal range 11.5–15.0 g/dL), accompanied by a decrease in serum albumin to 29.4g/L (normal range 35–52 g/L).Other laboratory test results, such as plasma concentrations of urea, creatinine, amylase, platelet counts, and coagulation, were within the normal range. Bedside colonoscopy found a large amount of bright red blood and blood clots in the left colon, and yellow-colored stool in the right colon. After careful washing, pulsatile fresh bleeding from an exposed vessel without a mucosal defect or ulceration was found 3 cm proximal to the anal verge (Fig. 1A). The lesion was treated with 3 hemostatic clips under transparent cap-assisted endoscopy (Fig. 1B–D). Follow-up colonoscopy after 3 days showed that the lesion had stabilized without further bleeding although 2 clips had dropped off. The patient’s condition improved and she was discharged 10 days later, and there was no bleeding after 3 months of follow-up.

### 3.2. Case 2

**Figure 2.**
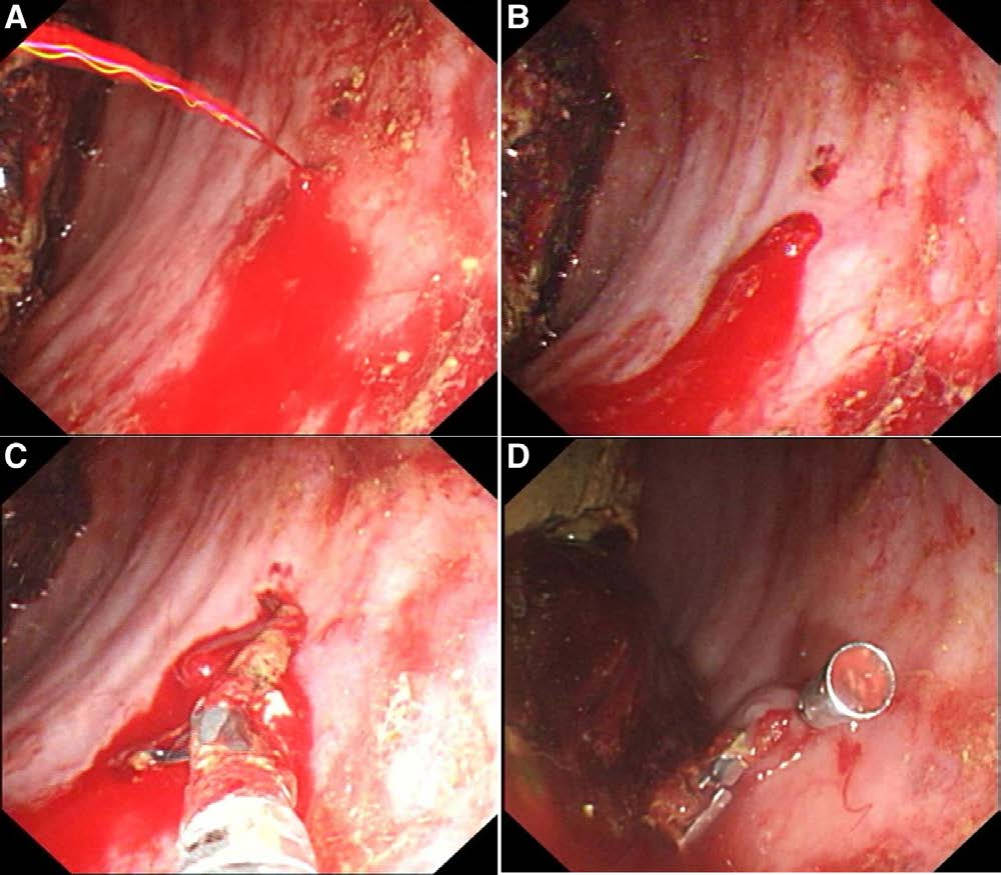
Endoscopy of patient 2. Bedside colonoscopy revealed a rectal Dieulafoy’s lesion at 5 cm from anal verge with active pulsating bleeding without surrounding ulceration (A, B). Two hemostatic clips were deployed to the Dieulafoy lesion, achieving successful hemostasis (C, D).

A 63-year-old man was admitted to the respiratory intensive care unit of our hospital due to fever with altered conscious level for a week. This patient was diagnosed with advanced lung cancer that had metastasized to multiple organs one month prior and had been administered durvalumab in combination with etoposide and carboplatin. He had a medical history of hypertension and constipation. After admission, the patient was administered noninvasive ventilator assisted ventilation and antibiotics, as well as diclofenac sodium suppositories and intravenous methylprednisolone sodium succinate injection. On the third day of admission, he had an attack of profuse hematochezia, his blood pressure dropped to 86/56 mmHg, and his hemoglobin level decreased from 13.1 to 9.1 g/dL (normal range 13–17.5 g/dL). Bedside colonoscopy revealed rectal Dieulafoy’s lesion 5 cm from the anal verge with active pulsating bleeding without surrounding ulceration (Fig. 2A and B). Immediate hemostasis was achieved by endoscopic therapy with 2 hemostatic clips (Fig. 2C and D and Supplemental Digital Content, Video, http://links.lww.com/MD/I14). The patient improved and was discharged 2 days later. There was no further bleeding after 1 month of follow-up.

## 4. Discussion

Dieulafoy’s lesions are characterized by aberrantly enlarged submucosal arterioles protruding through a small mucosal defect, which is an uncommon but well-recognized cause of acute life-threatening gastrointestinal hemorrhage.^[10]^ This lesion is majorly detected in the proximal stomach and is rarely reported in the rectum. Due to its rarity, the understanding of rectal Dieulafoy’s lesions mostly depends on case reports and small case series: there is a lack of systematic studies on its etiology, patient characteristics, clinical features, and therapeutic modalities.

We conducted a systematic literature search of the Pubmed database from 1991 to 2022 for rectal Dieulafoy’s lesions. A total of 64 cases were identified after careful review. Although Meister et al reported 2 cases in children in 1998,^[11]^ rectal Dieulafoy’s lesions frequently involve patients over the age of 60 (Table 1), with an average age of 68 years (range, 18–89 years). This lesion has a slight male predominance, with 59% of cases reported in men (Table 1). Patients with rectal Dieulafoy’s lesions commonly present with abrupt massive bright-red blood per rectum, hematochezia, or painless rectal bleeding, and the majority of patients experience sudden hemodynamic compromise. Similarly, one of our 2 cases involved rectal hemorrhage following subarachnoid hemorrhage surgery, and the other involved rectal hemorrhage during treatment for lung cancer and pulmonary infection.

**Table 1:** Clinical features for patients of rectal Dieulafoy’s lesion in this review.

Clinical features	N (%)
Gender	
Male	38 (59)
Female	26 (41)
Age	
0–19	3 (5)
20–39	2 (3)
40–59	10 (16)
60–79	36 (56)
80–99	13 (20)
Comorbid condition	
Hypertension	18 (28)
Chronic renal disease	14 (22)
Diabetes mellitus	13 (20)
Cardiovascular and cerebrovascular diseases	12 (19)
Cancer	6 (9)
Constipation	4 (6)
Dementia	3 (5)
Hyperlipidemia	2 (3)
Hyperuricemia	2 (3)
Diverticulosis	2 (3)
Place of onset	
In-hospital	24 (38)
Out-hospital	40 (62)

The pathogenesis of rectal Dieulafoy’s lesions is still unclear. Congenital and acquired vascular malformations have also been supposed to be plausible etiological factors for rectal Dieulafoy’s lesions.^[12]^ In this review, hypertension (28%), chronic renal disease (22%), diabetes mellitus (20%), cardiovascular and cerebrovascular diseases (19%), and cancer (9%) were among the most common comorbid conditions (Table 1). These conditions are all related to vascular abnormalities, which supports the vascular abnormality theory for rectal Dieulafoy’s lesion. Notably, 38% of patients in these cases were hospitalized with other conditions (Table 1), and the 2 patients in our case report were hospitalized due to severe complications. This has led to the suggestion of stress injury and predilection for the infirm.^[12]^ Nonsteroidal anti-inflammatory drugs, aspirin, and warfarin are used in approximately half of the patients with Dieulafoy’s lesions.^[13]^ The 2 patients in our study were administered aspirin, clopidogrel bisulfate, diclofenac sodium suppositories, and intravenous dexamethasone injections. Therefore, drug-induced mucosal injury might be one of the causes of Dieulafoy’s lesion rupture and bleeding;^[14,15]^ however, there is little evidence in the literature to support this. Mechanical pathologies such as constipation and fecalith have also been considered risk factors for rupture in these patients.^[13,16]^ Burns, dementia, hyperlipidemia, hyperuricemia, diverticulosis, and deep venous thrombosis are rare comorbidities of rectal disease; however, the association between these diseases requires further research.

The diagnosis of rectal Dieulafoy’s lesions is challenging in certain cases. This is mainly due to the small focus, relatively inconspicuous nature, and usually intermittent hemorrhage. Colonoscopy is the gold standard and the initial modality of choice in this situation due to its widespread availability and ease of use.^[12]^ The first patient in our report had a micropulsatile stream and the second patient had an arterial spurting through normal surrounding mucosa, which is consistent with the typical endoscopic appearance of rectal Dieulafoy’s lesion.

There is no consensus on the treatment of rectal Dieulafoy’s lesion. Therapeutic endoscopy has emerged to be a feasible, safe, and effective modality with a hemostatic success rate above 90%.^[17]^ Endoscopic therapy procedures can be classified into 3 groups according to the hemostatic mechanism^[12]^: thermocoagulation, which includes electrocoagulation, heat probe coagulation, and argon plasma coagulation; injection, which includes local epinephrine injection and sclerotherapy and mechanical hemostatic method, which includes banding and hemostatic clipping. In our review, endoscopic treatments of rectal Dieulafoy’s lesions were performed 69 times, and surgical ligation and angiographic embolization were both performed 5 times (Table 2). Thermocoagulation and injection, which were mostly reported before 2000 for rectal Dieulafoy’s lesion had similar primary hemostasis (75–80%) and rebleeding rate (25–30%) (Table 2). Endoscopic hemostatic clipping alone compared with band ligation alone achieved a higher primary hemostasis rate (100% vs. 83%) and a lower rebleeding rate (14% vs 25%) (Table 2). Therefore, endoscopic hemostatic clipping shows a slight therapeutic superiority over banding, thermocoagulation, and injection in rectal Dieulafoy’s lesions. Soetikno et al used an over-the-scope clip in the treatment of 5 patients with severe bleeding from the transitional zone of the anorectum, including from Dieulafoy’s lesion. Primary hemostasis was achieved in all the patients using one single over-the-scope clip, and no immediate or late rebleeding was observed.^[18]^ Combined endoscopic therapies were found to have a higher primary hemostasis rate (96% vs 88%) and a lower rebleeding rate (18% vs 22%) than those of endoscopic monotherapy (Table 2). It is worth mentioning that the epinephrine plus electrocoagulation or epinephrine plus heater probe in these reports achieved satisfactory primary hemostasis (100%) and rebleeding (7%) rates, which were comparable to those of endoscopic mechanical therapy (Table 2). However, these methods were mostly reported in the previous century, and few studies have been reported in recent years. However, mechanical endoscopic techniques appeared to be more feasible and were used in a majority of patients with rectal disease. Future research should compare the different methods, especially the effectiveness of hemostatic clipping and band ligation for rectal Dieulafoy’s lesion in large patient cohorts to determine the best endoscopic choice.

**Table 2:** Efficacy of different therapeutic modalities for rectal Dieulafoy’s lesion.

Therapeutic modality	Primary hemostasis rate n [%]	Rebleeding rate n [%]
Thermocoagulation	3/4 [75]	1/4 [25]
Injection	8/10 [80]	3/10 [30]
Mechanical hemostatic method		
EBL	10/12 [83]	3/12 [25]
EHP	14/14 [100]	2/14 [14]
OTSC	1/1 [100]	0 [0]
Combination endoscopic therapy		
Injection + thermocoagulation	15/15 [100]	1/15 [7]
Injection + EHP	8/9 [89]	3/9 [33]
Injection + EBL	3/3 [100]	1/3 [33]
Thermocoagulation + EBL	1/1 [100]	0 [0]
TAE	4/5 [80]	2/5 [40]
Surgical ligation	4/5 [80]	1/5 [20]

EBL = endoscopic band ligation, EHP = endoscopic hemoclip placement, OTSC = over-the-scope clip, TAE = transcatheter arterial embolization.

In conclusion, rectal Dieulafoy’s lesion represents a rare cause of lower gastrointestinal bleeding. Massive hemorrhage could increase the morbidity and mortality of these patients. We report 2 cases of acute hemorrhages due to rectal Dieulafoy’s lesion in 2 critically ill patients hospitalized for other conditions. Both patients were treated successfully by endoscopic hemostatic clipping, without rebleeding during follow-up. We propose that hemostatic clipping is one of the options in the treatment of rectal Dieulafoy’s lesions.

## Author contributions

**Conceptualization:** Ping Han, Wei Yan.

**Data curation:** Yu Lei.

**Investigation:** Ping Han, Wei Hou, Jingmei Liu.

**Methodology:** Yu Lei, Nianjun Chen.

**Resources:** Dean Tian, Qiaozhen Guo.

**Visualization:** Jingmei Liu, Dean Tian.

**Writing—original draft:** Ping Han, Yu Lei.

**Writing—review and editing:** Qiaozhen Guo, Wei Yan.

## Supplementary Material


